# The GLAaS algorithm for portal dosimetry and quality assurance of RapidArc, an intensity modulated rotational therapy

**DOI:** 10.1186/1748-717X-3-24

**Published:** 2008-09-09

**Authors:** Giorgia Nicolini, Eugenio Vanetti, Alessandro Clivio, Antonella Fogliata, Stine Korreman, Jiri Bocanek, Luca Cozzi

**Affiliations:** 1 Oncology Institute of Southern Switzerland, Medical Physics Unit, Bellinzona, Switzerland; 2 University of Lausanne, Faculty of Medicine, Lausanne, Switzerland; 3 University of Milan, Medical Physics Specialisation School, Milan, Italy; 4 Rigshospitalet, Radiation Oncology Dept, Copenhagen, Denmark; 5 Varian Medical Systems Int. AG, Zug, Switzerland

## Abstract

**Background:**

To expand and test the dosimetric procedure, known as GLAaS, for amorphous silicon detectors to the RapidArc intensity modulated arc delivery with Varian infrastructures and to test the RapidArc dosimetric reliability between calculation and delivery.

**Methods:**

The GLAaS algorithm was applied and tested on a set of RapidArc fields at both low (6 MV) and high (18 MV) beam energies with a PV-aS1000 detector. Pilot tests for short arcs were performed on a 6 MV beam associated to a PV-aS500. RapidArc is a novel planning and delivery method in the category of intensity modulated arc therapies aiming to deliver highly modulated plans with variable MLC shapes, dose rate and gantry speed during rotation. Tests were repeated for entire (360 degrees) gantry rotations on composite dose plans and for short partial arcs (of ~6 or 12 degrees) to assess GLAaS and RapidArc mutual relationships on global and fine delivery scales. The gamma index concept of Low and the Modulation Index concept of Webb were applied to compare quantitatively TPS dose matrices and dose converted PV images.

**Results:**

The Gamma Agreement Index computed for a Distance to Agreement of 3 mm and a Dose Difference (ΔD) of 3% was, as mean ± 1 SD, 96.7 ± 1.2% at 6 MV and 94.9 ± 1.3% at 18 MV, over the field area. These findings deteriorated slightly is ΔD was reduced to 2% (93.4 ± 3.2% and 90.1 ± 3.1%, respectively) and improved with ΔD = 4% (98.3 ± 0.8% and 97.3 ± 0.9%, respectively). For all tests a grid of 1 mm and the AAA photon dose calculation algorithm were applied. The spatial resolution of the PV-aS1000 is 0.392 mm/pxl. The Modulation Index for calculations resulted 17.0 ± 3.2 at 6 MV and 15.3 ± 2.7 at 18 MV while the corresponding data for measurements were: 18.5 ± 3.7 and 17.5 ± 3.7. Partial arcs findings were (for ΔD = 3%): GAI = 96.7 ± 0.9% for 6° rotations and 98.0 ± 1.1% for 12° rotations.

**Conclusion:**

The GLAaS method can be considered as a valid Quality Assurance tool for the verification of RapidArc fields. The two implementations (composite rotation or short arcs) allow the verification of either the entire delivery or of short partial segments to possibly identify local discrepancies between delivery and calculations. RapidArc, according to the findings, appears to be a safe delivery method in terms of dosimetric accuracy allowing its clinical application.

## 1. Background

Electronic portal imagers based on amorphous silicon flat panels are quite largely utilized for dosimetric purposes [1-7], mainly for pre-treatment IMRT verification beams, allowing time sparing and good accuracy. Performances and characteristics of the amorphous silicon detectors have been investigated. In particular, solid results exist on linear response in dose, non reproducibility of the off-axis ratio, different response at different field sizes and different energies and spectra, etc. In general, to manage undesired aspects of these detectors, special algorithms have been developed and adopted aiming to convert raw images into dose readings.

Our group developed one similar algorithm, named GLAaS [8,9] to convert PV-aS500 (and PV-aS1000) images into dose matrices. The starting point for GLAaS development was pre-treatment IMRT verifications and as such GLAaS is routinely used and results were reported. GLAaS is based on the application at pixel level of specific dose response parameters distinguishing between primary and transmitted radiation (from MLC or main jaws). In addition, GLAaS accounts for perturbations (e.g. the backscattering from the support arm or the beam over-flattening induced by the detector calibration for imaging) with some specific correction factors. GLAaS was recently further developed [10] to be used for dosimetric Quality Assurance of linear accelerators (e.g. to measure beam profiles for open and wedged, symmetric and asymmetric fields or to measure output and wedge factors for constancy checks).

The present report describes a new application of GLAaS for dosimetric verification of intensity modulated arcs. Recently, a novel technology called RapidArc was introduced on Varian linear accelerators. RapidArc belongs to the class of intensity modulated arc therapies [11-15]. This new delivery modality created the need of developing appropriate approaches to machine and pre-treatment verification processes. In literature so far, few publications addressed the usage of two-dimensional arrays to verify (modulated) arc fields. The usage of a 2D ion chamber array with a phantom with an octagonal shape was described in [16]. Other detectors have been used and tested in the framework of the RapidArc development teams and provided excellent results with the possibility to develop different verification strategies. In general, using external devices, particular attention shall be put to spatial resolution (some detectors have resolution coarser than 5 mm) and/or the usage of complementary phantoms. GLAaS constitutes an alternative to these approaches. It has some potential factors of interest being based on a detector (the PV-aS1000) available on RapidArc machines; it does not require any phantom for its application and it operates converting images into absolute dose matrices.

A possible limitation of GLAaS that has to be anticipated. Given the mechanical mounting of the detector, integral with the gantry, the arc verification is performed collapsing the entire rotation onto a single verification plane, creating a sort of composite dosimetric measurement. This feature could eventually mask the, unlikely, event of destructive interference of independent delivery (or calculation) errors. To overcome this feature, a pilot study is described in this report aiming to use GLAaS in a sort of fine-angular resolution mode by means of consecutive acquisition of short arc of few degrees in independent shots to be individually analysed in sequence.

## 2. Methods

### RapidArc technique

RapidArc is a novel planning and delivery technique for volumetric delivery of intensity modulated arcs, based on the concept as published by K. Otto [17]. It consists on a single arc where MLC (max 5 mm/degree and 2.5 cm/s), dose rate (max 600 MU/min), and gantry speed (max 72 s/turn, i.e. ~5 degrees/s) are optimized simultaneously to achieve the desired degree of modulation. At planning level, RapidArc consists of optimizing a dose distribution from dose-volume objectives including in the optimization the main characteristics of the linac head and the MLC (e.g. speed, transmission, rounded leaf tip and tongue and groove design). The entire gantry rotation is described in the optimization process by a sequence of 177 control points, CP, (one CP every roughly 2° of rotation). The final dose calculation is performed in Eclipse by means of the AAA algorithm.

One key point of RapidArc planning and delivery is the usage of collimator angles different from zero, typically in the range of 35–45 degrees. A non zero collimator angle implies that the tongue and groove effect, minimised by the optimizer but not completely avoided, is smeared out into non coplanar trajectories rather then being piled up in ''rings" orthogonal to the patients axis as if collimator would be set to zero.

The software version of both optimizer and dose calculation is a pre-clinical release of Eclipse 8.2.16. At delivery level, RapidArc plans are transferred by DICOM-RT communication to the 4D treatment console of the Varian linacs. Here, the actual treatment parameters are determined and transferred to the various system controllers. Particularly, the MLC controllers verifiy every 50 msec the position of the leaves with respect to expected, previous and following positions as well as the agreement of delivered dose. The linac controllers check, with the same frequency and logic, the angular position of the gantry and the dose rate. Whatever discrepancy should be detected by the controllers would generate immediate beam off interlock and the delivery would be interrupted.

### GLAaS algorithm

The GLAaS algorithm [8,9] has been used to convert raw images acquired with the portal imager into dose matrices at the depth of the maximum dose d_max_. No phantom is used, and radiation field impinges directly onto the detector. This algorithm was originally developed for IMRT pre-treatment verification, and here slightly adapted for RapidArc testing. A brief description of the algorithm follows.

For a given beam, the response of the amorphous silicon detectors is linear (*D*(*Gy*) = m*R+*q*). IMRT and RapidArc fields are, however, changing continuously during delivery. GLAaS accounts for those changes in time and position, using different *m *and *q *values, and differentiating between primary and transmitted (below the MLC) radiation, on a pixel by pixel basis.

The total dose *d*_*i *_in the *i-th *pixel, over the entire field delivery is:

(1) $$d_{i}=d_{pr,i}+d_{tr,i}=\left(\sum_{s=1}^{N}m_{pr,s}(EwwF)\cdot r_{i,s}+q_{pr,s}\right)+\left(m_{tr}\cdot \left(R_{i}-\sum_{s=1}^{N}r_{i,s}\right)+q_{tr}\right)$$

where: *m *and *q *are the slope and the intercept for a field of size *EwwF *(Equivalent window width Field), *r *is the reading attributed to the primary radiation for the segment/control point *s*, and *R *is the total PV reading; subscripts *pr *refer to primary, *tr *to transmitted radiation. The field is considered as a sum of *N *segments or control points. In the case of single static field or RapidArc, the key elements for GLAaS are the same: knowledge of the MLC shape and of the dose progress at any instant of the delivery. This information is fully stored in the DICOM-RT plans from the treatment planning system. In addition, RapidArc is characterised by variable dose rate during delivery. It was proven in [10] that the detector response is independent on the dose rate; in this view the same calibration parameters set can be used for the whole field, acquired at any (variable) dose rate.

The parameter values computed during the configuration of the GLAaS to analytically obtain the slopes come from the following empirical algorithm:

(2) *OF*(*EwwF*) = [*x *+ *d*·1n(*EwwF*)]^-1^

where *EwwF *is the equivalent field size of each segment

(3) *m*_*pr *_(*OF*) = *a*·*OF *+ *b*

where *m*_*pr *_is the slope for primary radiation, and *OF *is the PV measured output factor as per equation (2).

For transmitted radiation the following relationship is used:

(4) *m*_*tr *_= *k*·*m*_*pr*_

GLAaS configuration consists in the determination of a set of empirical parameters: *a, b, c, d, k, q*_*pr *_and *q*_*tr*_.

GLAaS has been configured to convert images acquired without any buildup on the PV cassette into dose at the depth of maximum dose d_max _(1.5 cm and 3 cm for 6 and 18 MV respectively), at the source-detector distance SDD = 100 cm.

The GLAaS algorithm was already tested [10] for verification of fields with high doses, needed when RapidArc fields are concerned, because the full dose is delivered in only one field. This is guaranteed by the way the PV electronics works, averaging the reading per each pixel over a number of frames, and recording the reading values and the number of acquisition frames.

### The equipment

To test the new RapidArc approach with GLAaS, a treatment unit installed at Rigshospitalet in Copenhagen has been used. It is a Clinac 2100iX, equipped with a Millennium multileaf collimator MLC-120, two photon energies of 6 and 18 MV, Portal Vision PV-aS1000 with full resolution (0.392 mm/pxl) and with Exact-arm support. The system allowed RapidArc delivery through a preclinical software release (vers. 8.2.13) that was installed during the month of February 2008 to perform delivery investigations.

The relevant acquisition parameters were: Acquisition Technique = Integrated Image, Readout = Sync-Integrated. The pilot study on short arcs was instead performed on a 6 MV beam from a Clinac 6 EX equipped with a PV-aS500 installed at the Oncology Institute of Southern Switzerland. This was necessary since the linac in Copenhagen was not further available for tests. Tests of short arcs were therefore performed on a simplified RapidArc model keeping both the dose rate and the gantry speed constant.

One preliminary test that was performed aimed to determine the eventual apparent PV displacement during gantry rotation. This could be to a true support arm mechanical instability or to gantry sag. The test was exploited measuring during entire arcs of 360°, clockwise and counterclockwise, 'cine mode', i.e. with a sequence of 240 images acquired during the arc, a small field of 0.4 × 0.4 cm^2^. The centres of mass of all images were recorded, and differences with respect to the gantry at 180° (the starting position) were analysed in *x *and *y *directions and for both rotation directions.

#### GLAaS applied to RapidArc plans

Seven 'clinical' deliveries, i.e. from true RapidArc plans optimized for test patients were selected from various tumour sites (brain, head and neck, thorax, and pelvis). Different dose prescriptions were applied (from 1.2 to 2.5 Gy/fraction, some including Simultaneous Integrated Boost). Cases were selected to guarantee a variety of PTV volumes (ranging from 110 to 1060 cm^3^). This wide variability was chosen to asses the GLAaS performances on various RapidArc plan possibilities. Plans were delivered for both 6 and 18 MV beams. MLC, dose rate and gantry rotation were operated during the delivery as required by the clinical plan generating a composite dose image on the detector that rotates integral with the gantry.

Dose calculations for GLAaS comparison were obtained from Eclipse. For each RapidArc plan, a "verification plan" was generated on a water phantom, using the original parameters (MLC and dose rate). This verification plan was then "collapsed" on an infinitesimal gantry rotation, generating a dose distribution of the whole arc on a single plane, orthogonal to the beam central axis as the detector. The calculated dose matrix therefore, as the measurement, integrated the dose contributions delivered at various angular positions into one single plane. The dose calculations in the phantom were based on the same algorithm implemented for RapidArc plans. Every control point was individually calculated and accumulated, computing the dose distribution for each CP separately. This allowed to build a verification plan equivalent to the actual patient plan. In this study, dose distributions were exported from Eclipse at d_max _and isocentre distance (given the SDD = 100 cm). Dose calculation grid of verification plans was set to 1 mm, being the minimum allowed with AAA dose calculation.

Measured matrices at d_max _converted into dose through GLAaS were compared to the corresponding computed doses. Evaluation was performed via the gamma index [18] on the field area defined by the jaw setting. The evaluation criteria were chosen as follows: DTA (distance to agreement) of 3 mm and ΔD (dose difference) of 2, 3 and 4%. ΔD was defined respect to the significant maximum dose of the field. This was defined as [8] the maximum dose value in the distribution of measured dose in the field after cutting the highest 5% dose tail in the histogram. The Gamma Agreement Index (GAI), defined as the percentage of points inside the field passing the gamma evaluation criteria, was computed for each case, and then averaged over all the cases.

To further analyse their agreement, the Modulation Index MI [19,20] was computed for both measured and calculated dose matrices for each field. As known, this parameter is a measure of the degree of modulation:

$$MI(F)=\int_{0}^{F}Z(f)df$$

Where Z(f) is the spectrum of the modulation pattern in the field and the integration limit F was set to 1, as described in [20]. The application of the MI on the dose matrices instead of the fluence maps, allows a more direct evaluation of the 2D dose maps to appreciate e.g. eventual dependencies on the dose calculation grid. In this respect, a comparison of the MI for measured and calculated doses results in an appreciation of the relative compatibility between calculation grid and detector resolution.

#### Partial arcs

A pilot study was performed to assess the GLAaS capability to detect fine and local features of RapidArc dose calculation with respect to delivery. This was performed computing and measuring, at various positions during the arc rotation, some short consecutive partial arcs. For two of the seven plans, three subsequent sub-arcs of ~6° (178.0–171.8°, 171.8–165.7°, 165.7–159.6°) and of ~12° (178–165.7°, 165.7–153.4°, 153.4–141.2°) were analysed. In these cases, interrupted plans were generated in Eclipse selecting the starting and ending control points (4 CP corresponding to 3 intervals for the 6° sub-arcs, 7 CP corresponding to 6 intervals for the 12° sub-arcs). Verification plans were computed with the same algorithm used for the entire arc plan. Interrupted plans were used also to acquire images. These tests were performed with fixed dose rate and fixed gantry speed on the 6 MV beam of the IOSI linac.

Analysis with the gamma evaluation was performed on the minimum rectangular field area that includes the sub-arc (instead of the jaws defined field) in order to have a fair comparison between full arc and partial arc. From gamma evaluation analysis the GAI were recorded, with the criteria of DTA = 3 mm and ΔD = 3%.

For this pilot study, the short term stability of repeated deliveries was tested through three consecutive measurements of each sub-arc.

## 3. Results

**Figure 1:**
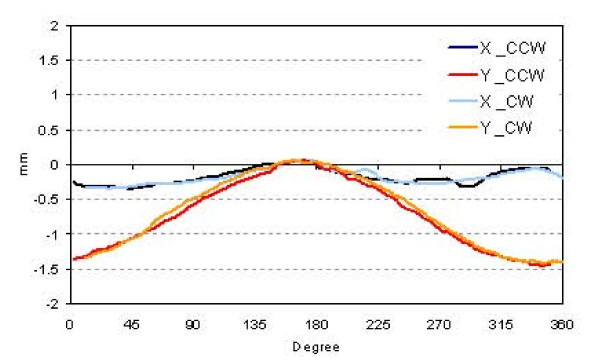
Apparent PV displacement relatively to the centre of rotation for a small field during full rotation in both directions (*x *and *y*), for clockwise and counter clockwise rotations.

The results of the assessment of the apparent PV support arm movement are reported in figure 1 where the displacement of the center of mass of the small field is plotted as a function of the gantry position. Blue and light blue lines, that refer to the displacement in the transversal direction (*x*), present a small movement within 0.3 mm. Red and orange lines relate to the longitudinal displacement (*y *direction): here the movement is larger, showing a maximum displacement of -1.5 mm around the gantry 0° position with respect to the 180° position.

### GLAaS applied to RapidArc plans

**Figure 2:**
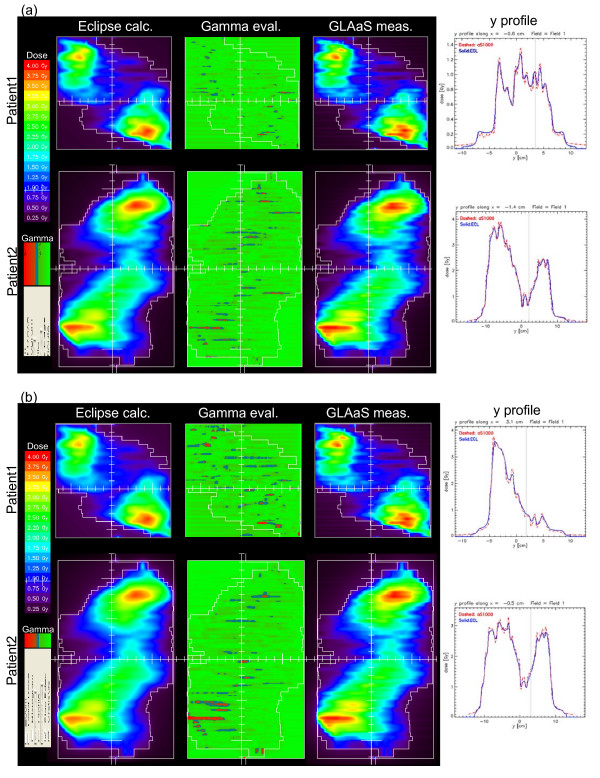
Examples of QA for RapidArc plans. Columns refer to: Eclipse dose map, gamma evaluation map, GLAaS dose map, and profile in a longitudinal direction. (a) 6 MV, (b) 18 MV.

In figure 2 two examples of the analysis of one RapidArc field relatively to the measured GLAaS dose image are shown for 6 MV (a) and 18 MV (b). In the first column, the computed (Eclipse) dose maps are shown. In the second column the gamma maps are reproduced. The GLAaS dose matrices are reproduced in the third column while in the last column, an example of profile comparisons in the *y *direction is presented. It can be seen how collapsing the entire delivery onto a single plane introduces back into the delivery the tongue and groove pattern that would be smeared out (and almost cancelled) in the clinical case.

Summary of the results of the full arc deliveries is presented in table 1, with the GAI averaged over all the seven plans (DTA = 3 mm, ΔD = 3%), as well as the MI for the calculated and measured dose maps, for both 6 and 18 MV. GAI was better than 95% for all cases at 6 MV and in average for 18 MV (with ~93% as minimum agreement). The MI of the calculated dose map is lower than the MI for measurements of about 8% for 6 MV, and the difference is statistically significant (p = 0.001 with a paired t-test). As a confirmation of the influence of calculation grid (or detector resolution), the average MI of dose maps at 6 MV computed with a 2.5 mm calculation grid is 15.9 ± 2.7 (instead of 17.0 at 1 mm). This value is 14% lower than the MI from GLAaS and corresponds to smoother dose calculations if compared to the 1 mm case. This feature would be reflected also in lower GAI if 2.5 mm calculations would be used for comparison. In this latter case the average GAI (6 MV) was 93.9 ± 2.4% with a minimum of 91.6%.

**Table 1:** Summary of the GLAaS results in terms of GAI and MI.

Energy		GAI [%]	MI calc	MI GLAaS
6 MV	Mean	96.7 ± 1.2	17.0 ± 3.2	18.5 ± 3.7
	Range	[95.3, 98.5]	[11.4, 20.3]	[11.9, 22.2]
18 MV	Mean	94.9 ± 1.3	15.3 ± 2.7	17.5 ± 3.7
	Range	[92.9, 96.2]	[11.3, 18.8]	[11.8, 21.8]

Evaluation of the data with different ΔD is reported in table 2. For ΔD = 2% the agreement is, as expected poorer, but it shall also be noticed that in this case value of the SD is higher while it decreases when increasing ΔD. ΔD thresholds should therefore be tuned also to balance between desired precision and noise.

**Table 2:** Summary of the GLAaS results: GAI values for different threshold criteria.

Energy	GAI [%] DTA, ΔD = 3 mm,2%	GAI [%] DTA, ΔD = 3 mm,3%	GAI [%] DTA, ΔD = 3 mm,4%
6 MV	93.4 ± 3.2	96.7 ± 1.2	98.3 ± 0.8
18 MV	90.1 ± 3.1	94.9 ± 1.3	97.3 ± 0.9

### Interrupted arcs

**Figure 3:**
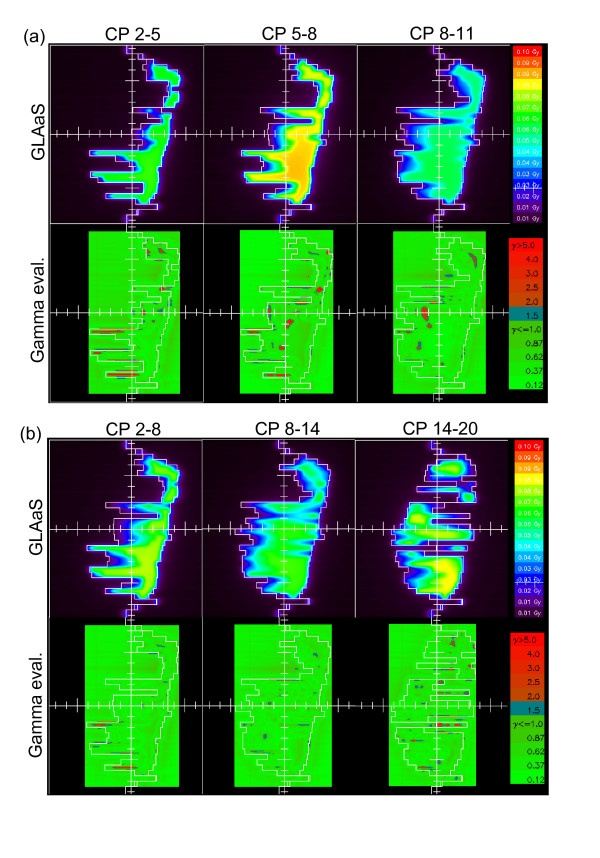
GLAaS dose and gamma evaluation maps for the three consecutive sub-arcs of: (a) 6 degree, (b) 12 degree.

Figure 3 shows the GLAaS dose (first row) and the gamma evaluation matrix (second row) for one plan and the three sub-arcs for the 6 and 12° gantry movement.

In table 3 the GAI results are reported as mean values and standard deviations of all the acquisitions per each gantry interval. Also the mean values and standard deviations, as well as the range, are reported as global mean of all the 6° and 12° sub-arcs (both patients, the three acquisitions per sub-arc). Those values, both for 6 and 12° sub-arcs (GAI = 97 and 98%), are consistent with the corresponding full arcs (GAI = 97%).

**Table 3:** Summary of the interrupted arc analysis

Arc interval	GAI [%] mean ± SD	GAI [%] Range
178.0–171.8°	96.0 ± 0.7	
171.8–165.7°	97.0 ± 0.8	
165.7–159.6°	97.2 ± 0.7	
**6°**	**96.7 ± 0.9%**	**[95.1, 98.0]**

178.0–165.7°	98.6 ± 0.5	
165.7–153.4°	97.8 ± 0.2	
153.4–141.2°	96.6 ± 0.9	
**12°**	**98.0 ± 1.1%**	**[95.6, 99.0]**

The results show better figures for larger arc intervals for two reasons. Firstly the low number of MU for very small arcs (in average 11 MU for 6°, 21 MU for 12° arcs) enhances the uncertainty in measurements. Secondly the highly jagged shape of the irradiated area, with regions presenting alternate open and closed leaves enhances the role of small discrepancies between measurements and calculations of leaf edge penumbra due to the different spatial resolutions.

**Figure 4:**
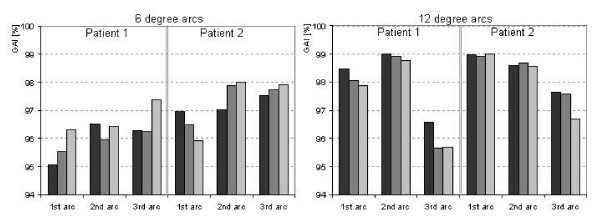
GAI results (DTA = 3 mm, ΔD = 3%) for all acquisition of sub-arcs findings showing repeated measurements.

Nevertheless, repeated measurements of small arcs resulted in reproducible findings. The maximum observed variation of the GAI during the three acquisitions was of 1.3%, with a mean variation of 0.7 ± 0.4%. This is a confirmation of the stability of the delivery, even in case of small arcs. Plots of the repeatability over the three acquisitions in all cases are shown in figure 4, where all the GAI values are reported.

## 4. Discussion and conclusion

The usage of GLAaS to perform RapidArc pre-treatment quality assurance, and in principle also any machine QA based on modulated arcs, was presented in the present report. RapidArc is a novel approach to intensity modulated arc therapy with linacs. With RapidArc, in addition to MLC based modulation, also the dose rate and the gantry rotation speed are varied during delivery to enhance the modulation degree. Principal aim of RapidArc is to deliver plans of high conformal avoidance with a single arc and minimal delivery time. Dedicated quality assurance processes are being developed by first RapidArc users in the Council of Developers and investigations on GLAaS belong to this category. The main benefits of GLAaS applied to RapidArc can be summarized as follows: *i) *GLAaS performances were proven for static field dynamic IMRT and for standard machine quality assurance [8-10]; *ii) *GLAaS makes usage of the PV-aS1000 (or PV-aS500), integrated in the delivery system and does not require complementary phantoms or external systems; *iii) *GLAaS allows to convert measured data into absolute doses at d_max _and to directly compare these against calculations from the planning system; *iv) *the spatial resolution of the PV-aS1000 is superior to other currently available system.

Concerning spatial resolution, it was shown in this report that care shall be put in selecting appropriate experimental settings to minimize perturbations. Particularly, if GLAaS dosimetry with the PV-aS1000 is used, the dose calculation grid in the planning system should be the finest possible. On the other hand, with GLAaS it is possible, in the composite and in the short arc modes, to identify eventual computational problems that are hardly detectable by other systems. The analysis of Modulation Index helped to investigate more the agreement between datasets and the relevance of calculation grid resolution. The observed difference in MI between calculation and delivery derives from two possible sources. A dose calculation grid of 1 mm could be still too coarse for a detector with ~0.4 mm spatial resolution (but 1 mm is the minimum value selectable in Eclipse). The creation of composite verification plans could introduce some bias (either smoothing or enhancing modulation) in the calculation or in the delivery. Nevertheless the difference observed in MI, even if statistically significant, was found to be small in absolute terms.

Some items are specific of arc based delivery and to the specific RapidArc implementation and should be addressed for GLAaS. Concerning arcs, the first problem was the assessment of the magnitude of the apparent displacement of the detector with respect to the rotational axis (due to both gantry sag and real detector mechanical instability). The movement along the transversal direction was considered as negligible since it is of the same magnitude of the PV pixel size. Along the longitudinal direction x, about one third of the whole rotation present a displacement inferior to 1–1.5 mm. This value is roughly one third of the penumbra of the lateral leaf's edge. These two reasons (only 30% of the delivery is affected and the correlation to penumbra size) allowed the decision to exclude from the GLAaS development for arc the need, at first order, of dedicated corrections for the apparent detector displacement with respect to the axis of rotation. Would a specific system require it, it would be possible to implement an off line correction. A feasibility test was performed by applying or not to GLAaS such a correction. The difference was negligible, and the GAI difference between the two cases was less than 0.5%.

A second item was linked to the usage of variable dose rate during delivery. This issue was not addressed in this report since it was already investigated for Enhanced Dynamic Wedges in [10]. In that report it was shown how GLAaS based dosimetry is independent from the dose rate due to the characteristics of the PV-aS detectors.

Onether potential limitation to mentione is that, whatever the collimator angle, being the detector integral with the gantry, the residual tongue and groove effect is brought back in the measurements as noticed in figures 2 and 3. This is likely the main contributor to the GAI deviation from 100% but, as results proved, it does not compromise the high quality of results. The presence of the tongue and groove patterns would be solved if the detector would be detached from the gantry and left fixed on the couch. Another issue to mention is that, RapidArc verification through composite planes does not allow any direct verification of the gantry rotation and that fluctuations linked to this are eventually lost. To minimize the relevance of this limitation, partial short arcs should be verified and included in the standard procedures. The application of GLAaS to short arcs would also basically solve the issue of tongue and groove effect (at least would not enhance it through its pile-up)

Notwithstanding the above limitations, the results summarised in this report are quite satisfactory. In the composite mode, average GAI equal or higher than 95% were obtained for both low and high energy modes and the range of findings never fell below 90%. These results are consistent with the current clinical experience from GLAaS applied to standard IMRT verification where, at low energy, an average GAI of ~98% is observed [9]. At low energy, the tests performed on short arcs showed similar results with GAI higher than 96% and with a highly reproducible pattern for repeated measures on the short term.

In absence of any general consensus on acceptance levels for modulated arc delivery, it is authors opinion that a threshold to GAI = 95% could be applied to define acceptable pre-treatment delivery verifications. For GAI in the range between 90 and 95% care should be paid to investigate more in detail potential sources of errors by performing complementary tests (e.g. controlling leaf motion, dose rate or gantry speed performances). GAI should likely be computed with DTA = 3 mm and ΔD = 3% since calculation uncertainties from TPS and detector vs. calculation spatial resolution issues would weaken reliability of findings optained with more stringent parameters.

To conclude, GLAaS can be considered as a promising approach to RapidArc delivery Quality Assurance in both the composite and short arcs approaches. Further development are needed to make the short arc more automatic but do not require changes to the model. At the same time, RapidArc delivery was tested for a variety of different indications and results are satisfactory and allow considering safe its clinical introduction.

## Competing interests

The authors declare that they have no competing interests. Dr Luca Cozzi acts as scientific consultant to Varian Medical Systems AG. Jiri Bocanek is a Varian Medical System employee.

## Authors' contributions

GN, AF and LC designed the study. LC wrote the manuscript. GN, EV, AC, SK, JB performed data acquisition and processing. AF, GN, LC, EV and AC developed the algorithms. EV, AC and GN wrote the computer programmes. All authors reviewed and approved the manuscript.

## References

[B1] Berger L, François P, Gaboriaud G, Rosenwald JC (2006). Performance optimization of the Varian aS500 EPID system. J Appl Clin Med Phys.

[B2] Greer PB, Popescu CC (2003). Dosimetric properties of an amorphous silicon electronic portal imaging device for verification of dynamic intensity modulated radiation therapy. Med Phys.

[B3] Greer PB (2007). Off-axis dose response characteristics of an amorphous silicon electronic portal imaging device. Med Phys.

[B4] Greer PB, Vial P, Oliver L, Baldock C (2007). Experimental investigation of the response of an amorphous silicon EPID to intensity modulated radiotherapy beams. Med Phys.

[B5] Grein EE, Lee R, Luchka K (2002). An investigation of a new amorphous silicon portal imaging device for transit dosimetry. Med Phys.

[B6] Parent L, Fielding AL, Dance DR, Seco J, Evans PM (2007). Amorphous silicon EPID calibration for dosimetric applications: comparison of a method based on Monte Carlo prediction of response with existing techniques. Phys Med Biol.

[B7] Winkler P, Hefner A, Georg D (2005). Dose-response characteristics of an amorphous silicon EPID. Med Phys.

[B8] Nicolini G, Fogliata A, Vanetti E, Clivio A, Cozzi L (2006). GLAaS: an absolute dose calibration algorithm for an amorphous silicon portal imager. Applications to IMRT verification. Med Phys.

[B9] Nicolini G, Fogliata A, Vanetti E, Clivio A, Vetterli D, Cozzi L (2008). Testing the GLAaS algorithm for dose measurements on an amorphous silicon portal imager on low and high energy photon beams. Med Phys.

[B10] Nicolini G, Vanetti E, Clivio A, Fogliata A, Boka G, Cozzi L (2008). Testing the portal imager GLAaS algorithm for machine quality assurance. Radiat Oncol.

[B11] Duthoy W, De Gersem W, Vergote K (2004). Clinical implementation of intensity-modulated arc therapy (IMAT) for rectal cancer. Int J Radiat Oncol Biol Phys.

[B12] Earl MA, Shepard DM, Naqvi S, Li XA, Yu CX (2003). Inverse planning for intensity-modulated arc therapy using direct aperture optimization. Phys Med Biol.

[B13] Wong E, Chen JZ, Greenland J (2002). Intensity-modulated arc therapy simiplified. Int J Radiat Oncol Biol Phys.

[B14] Yu CX (1995). Intensity modulated arc therapy with dynamic multileaf collimation: an alternative to TomoTherapy. Phys Med Biol.

[B15] Yu CX, Li XA, Ma L (2002). Clinical Implementation of intensity-modulated arch therapy. Int J Radiat Oncol Biol Phys.

[B16] van Esch A, Clermont C, Devillers M, Iori M, Huyskens DP (2007). On-line quality assurance of rotational radiotherapy treatment delivery by means of a 2D ion chamber array and the Octavius phantom. Med Phys.

[B17] Otto K (2008). Volumetric modulated arc therapy: IMRT in a single gantry arc. Med Phys.

[B18] Low DA, Harms WB, Mutic S, Purdy JA (1998). A technique for the quantitative evaluation of dose distributions. Med Phys.

[B19] Webb S (2003). Use of a quantitative index of beam modulation to characterize dose conformality: illustration by a comparison of full beamlet IMRT, few-segment IMRT (fsIMRT) and conformal unmodulated radiotherapy. Phys Med Biol.

[B20] Nicolini G, Fogliata A, Vanetti E, Clivio A, Ammazzalorso F, Cozzi L (2007). What is an acceptably smoothed fluence? Dosimetric and delivery considerations for dynamic sliding window IMRT. Radiat Oncol.

